# Blood-brain barrier damages and intrathecal synthesis of anti-N-methyl-D-aspartate receptor NR2 antibodies in diffuse psychiatric/neuropsychological syndromes in systemic lupus erythematosus

**DOI:** 10.1186/ar4518

**Published:** 2014-03-21

**Authors:** Shunsei Hirohata, Yoshiyuki Arinuma, Tamiko Yanagida, Taku Yoshio

**Affiliations:** 1 Department of Rheumatology and Infectious Diseases, Kitasato University School of Medicine, 1-15-1 Kitasato, Sagamihara, Kanagawa 252-0374, Japan; 2 Department of Internal Medicine, Teikyo University School of Medicine, Tokyo 173-8605, Japan; 3 Division of Rheumatology and Clinical Immunology, Jichi Medical University, Tochigi 329-0498, Japan

## Abstract

**Introduction:**

Although neuropsychiatric systemic lupus erythematosus (NPSLE) is one of the recalcitrant complications of the disease, its pathogenesis still remains unclear. Previous studies revealed that antibodies reactive with NMDA (N-methyl-D-aspartate) receptor NR2 (anti-NR2) are elevated in cerebrospinal fluid (CSF) of patients with diffuse psychiatric/neuropsychological syndromes (diffuse NPSLE), which is usually more recalcitrant than neurologic syndromes of NPSLE (focal NPSLE). Two mechanisms have been implicated for the elevation of CSF IgG, including intrathecal synthesis and transudation through the damaged blood-brain barrier (BBB). The present study was designed in order to elucidate the roles of BBB function and intrathecal synthesis of anti-NR2 in the elevation of CSF anti-NR2 with regard to the severity in NPSLE.

**Methods:**

Paired serum and CSF samples were obtained from 81 systemic lupus erythematosus (SLE) patients when they presented active neuropsychiatric manifestations, and from 22 non-SLE control patients with non-inflammatory neurological diseases. The 81 SLE patients consisted of 55 patients with diffuse NPSLE, including 23 patients with acute confusional state (ACS), the severest form of diffuse NPSLE, and 26 patients with neurologic syndromes or peripheral nervous system involvement (focal NPSLE). IgG anti-NR2 and albumin were measured by ELISA. BBB function and intrathecal synthesis of anti-NR2 were evaluated by Q albumin and by CSF anti-NR2 index, respectively.

**Results:**

CSF anti-NR2 levels, Q albumin and CSF anti-NR2 index were significantly higher in NPSLE than in non-SLE control. CSF anti-NR2 levels and Q albumin were significantly higher in ACS than in non-ACS diffuse NPSLE (anxiety disorder, cognitive dysfunction, mood disorder and psychosis) or in focal NPSLE, whereas there was no significant difference in CSF anti-NR2 index among the 3 groups. CSF anti-NR2 levels were significantly correlated with Q albumin in diffuse NPSLE (r = 0.3754, *P* = 0.0053).

**Conclusions:**

These results demonstrate that the severity of BBB damages plays a crucial role in the development of ACS, the severest form of diffuse NPSLE, through the accelerated entry of larger amounts of anti-NR2 into the central nervous system.

## Introduction

Neuropsychiatric systemic lupus erythematosus (NPSLE) is one of the recalcitrant complications of the disease, leading to substantial impairment of quality of life as well as disability [1,2]. Among a variety of manifestations in NPSLE, acute confusional state (ACS) in diffuse psychiatric/neuropsychological syndromes (diffuse NPSLE) is the most serious, requiring extensive immunosuppressive therapy and sometimes resulting in poor prognosis [3,4].

Several studies have demonstrated that IL-6 in cerebrospinal fluid (CSF) is elevated in patients with NPSLE, including those with diffuse NPSLE and focal NPSLE [5,6]. Among various cytokines, IL-6 and chemokines, but not Th1/Th2 cytokines, were found to be significantly elevated in NPSLE [7,8]. Notably, a multicenter retrospective study with SLE patients who showed psychiatric manifestations demonstrated that the sensitivity and specificity of CSF IL-6 for diffuse NPSLE were 87.5% and 92.3%, respectively [9]. CSF IL-6 appeared to be elevated independently of serum IL-6 [5,9]. It was also found that CSF IL-6 was not correlated with specific blood abnormalities, CSF cell counts, or abnormalities identified on brain magnetic resonance imaging and electroencephalography [10]. Thus, the mechanism of the elevation of IL-6 in CSF remains unclear.

On the other hand, previous studies showed that autoantibodies against neuronal cells were specifically elevated in CSF from patients with diffuse NPSLE [11,12]. Several antigens have been implicated for the candidates of the targets of autoantibodies against neuronal cells, including ribosomal P proteins [13]. Of note, recent studies have demonstrated that CSF antibodies reactive with N-methyl-D-aspartate (NMDA) receptor NR2 subunit on neuronal cells (anti-NR2) are associated with diffuse NPSLE [14-16]. However, neither the precise mechanism of the elevation of CSF anti-NR2 nor its relevance with the severity of diffuse NPSLE is understood.

Two mechanisms have been implicated for the elevation of CSF IgG, including intrathecal synthesis and transudation through the damaged blood-brain barrier (BBB) (more strictly, the blood-CSF barrier) [17-19]. Since intrathecal IgG production has been shown to be elevated in NPSLE [18,20], it is likely that intrathecal production of anti-NR2 might be enhanced in diffuse NPSLE. On the other hand, it is also possible that transudation of anti-NR2 through the damaged BBB might contribute to the elevation of anti-NR2 in CSF. However, the nature of the integrity of the BBB in NPSLE has not been fully delineated. The current studies examined the BBB function and intrathecal synthesis of anti-NR2 in relation to the severity of diffuse NPSLE. It should be pointed out that BBB refers to the morphological basis for restriction of protein entry from the systemic circulation into the brain tissue, particularly through the brain capillary walls [19]. As albumin is produced exclusively in the liver, the increase of albumin in CSF is considered to reflect damage of the BBB. Thus, the albumin CSF/serum concentration ratio, called Q albumin, is widely accepted as an indicator of BBB function [17,19].

## Methods

### Patients and samples

Eighty-one patients with SLE were included in the present study. All patients fulfilled the American College of Rheumatology (ACR) 1982 revised criteria for the classification of systemic lupus erythematosus (SLE) [21]. Of the 81 SLE patients, 55 had diffuse psychiatric/neuropsychological syndromes (diffuse NPSLE) according to the 1999 ACR definition of NPSLE [3], whereas 26 patients had neuropsychiatric manifestations other than diffuse NPSLE, including neurologic syndromes and peripheral nervous system involvement (focal NPSLE) (Table 1). Among the 55 patients with diffuse NPSLE, 23 patients had complications due to acute confusional state (ACS), the severest form of diffuse NPSLE [3]. All the patients with NPSLE were hospitalized in Teikyo University Hospital or other correlated hospitals between 1993 and 2007. In addition, 22 patients with non-SLE non-inflammatory neurologic diseases (8 with cervical spondylosis, 7 with cerebrovascular disease, 3 with neurodegenerative disease, two with hyperventilation syndrome, 1 with headache, and 1 with diabetic neuropathy) were studied as non-SLE controls. All the 103 patients gave informed consent, and the study was approved by the institutional ethical committee of Teikyo University School of Medicine. CSF specimens were obtained from the patients by a lumbar puncture on the same day that serum samples were obtained, when the diagnosis of NPSLE was made by neurologists and rheumatologists. These samples were kept frozen at -30°C until they were assayed. All assays were performed without knowledge of the diagnosis or clinical presentations. Furthermore, upon entering the present study, the diagnosis of the 81 patients with NPSLE and its classification was reconfirmed by hospital case records.

**Table 1:** Profiles of the patients studied

**Diagnosis**	**Patients (number)**	**Gender**	**Age**
		**(male/female)**	**(mean ± SD)**
**Systemic lupus erythematosus (SLE)**	81		
Diffuse neuropsychiatric-SLE	55	6/49	37.0 ± 14.2
Acute confusional state	23		
Anxiety disorder	3		
Cognitive dysfunction	8*		
Mood disorder	13		
Psychosis	8		
Focal neuropsychiatric-SLE	26	4/22	41.4 ± 15.1
Cerebrovascular disease	11		
Demyelinating syndrome	1		
Headache	3		
Movement disorder	2		
Seizure disorder	8		
Polyneuropathy	1		
**Non-SLE control**	22	21/1	48.0 ± 13.7

*Two patients also presented mood disorder.

### Measurement of albumin

Albumin in CSF and sera was measured by ELISA using Human Albumin ELISA Quantitation Set (Bethyl Laboratories, Montgomery, Tx, USA).

### Evaluation of BBB function and intrathecal synthesis of anti-NR2

BBB function and intrathecal synthesis of anti-NR2 were evaluated by Q albumin (CSF albumin × 1,000/serum albumin) and by CSF anti-NR2 index ((CSF anti-NR2 × serum albumin)/(serum anti-NR2 × CSF albumin)), respectively, as previously described [17-19].

### Measurement of autoantibodies to the N-methyl-D-aspartate (NMDA) receptor subunit NR2

Anti-NR2 in sera and CSF were determined by specific ELISA using the highly purified synthetic 10 amino-acid peptide DWEYSVWLSN [22], conjugated to human serum albumin (HSA) as previously described [14]. The concentration of anti-NR2 that produced half of the maximal absorbance at 492 nm, given by saturating concentrations of anti-NR2 in the ELISA plate, was arbitrarily defined as 1 U/ml. The specific anti-NR2 activities were determined by subtracting the values for the nonspecific binding activity to HSA from those for binding activity to NR2 peptide-HSA conjugates [14].

### Measurement of IL-6 and anti-ribosomal P

CSF IL-6 was measured by bioassay using IL-6-dependent cell line MH60.BSF2 [5]. Serum anti-ribosomal P was determined by ELISA using purified bovine ribosomal P proteins (Arotec, Wellington, New Zealand), as previously described [13].

### Statistical analysis

Differences in various parameters between control and NPSLE or among various groups of NPSLE were analyzed by the Mann-Whitney *U*-test or the Kruskal-Wallis test with the Dunn multiple comparison test, respectively, using GraphPad Prism 6 for Mac OS X ver. 6.0b, GraphPad Software, Inc., San Diego, CA, USA. Correlation between various parameters was examined by the Spearman rank correlation test.

## Results

### CSF anti-NR2 are correlated with the severity of NPSLE

**Figure 1:**
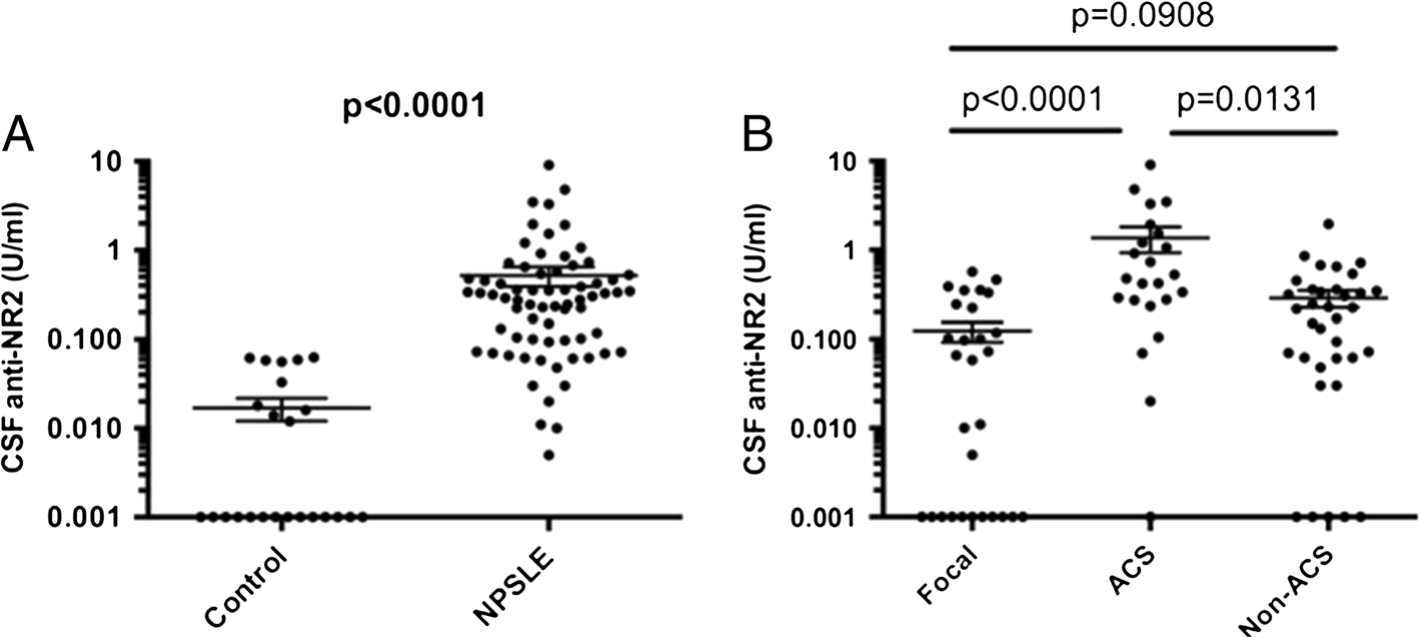
**Cerebrospinal fluid (CSF) antibodies to the N-methyl-D-aspartate (NMDA) receptor subunit NR2 (anti-NR2) in neuropsychiatric systemic lupus erythematosus (NPSLE). (A)** Comparison between NPSLE and non-SLE controls, **(B)** comparison among various subtypes of NPSLE. ACS, acute confusional state; Non-ACS, diffuse NPSLE other than ACS, including anxiety disorder, cognitive dysfunction, mood disorder and psychosis; Focal, focal NPSLE. Statistical analysis was performed by the Mann-Whitney *U*-test **(A)** and the Kruskal-Wallis test with the Dunn multiple comparison test **(B)**.

Initial experiments compared CSF anti-NR2 levels in various groups. As shown in Figure 1A, CSF anti-NR2 levels were significantly elevated in NPSLE compared with the non-SLE control group. NPSLE consists of focal NPSLE and diffuse NPSLE, including ACS or non-ACS (anxiety disorder, cognitive dysfunction, mood disorder and psychosis). CSF anti-NR2 levels were significantly elevated in ACS diffuse NPSLE compared with those in focal NPSLE and in non-ACS diffuse NPSLE (Figure 1B). CSF anti-NR2 levels appeared to be higher in non-ACS diffuse NPSLE than those in focal NPSLE, although it did not reach the statistical significance. The results indicate that CSF anti-NR2 levels are correlated with the severity of NPSLE in terms of ACS.

It is pointed out that psychosis is sometimes presented as a severe manifestation [3]. However, CSF anti-NR2 levels in eight patients with psychosis (0.145 ± 0.047 U/ml (mean ± SD)) were not significantly different from those in 24 patients with non-ACS non-psychosis diffuse NPSLE (0.271 ± 0.053 U/ml) (*P* = 0.3728 as evaluated by the Mann-Whitney *U*-test).

### Intrathecal anti-NR2 synthesis and BBB function in each subset of NPSLE

**Figure 2:**
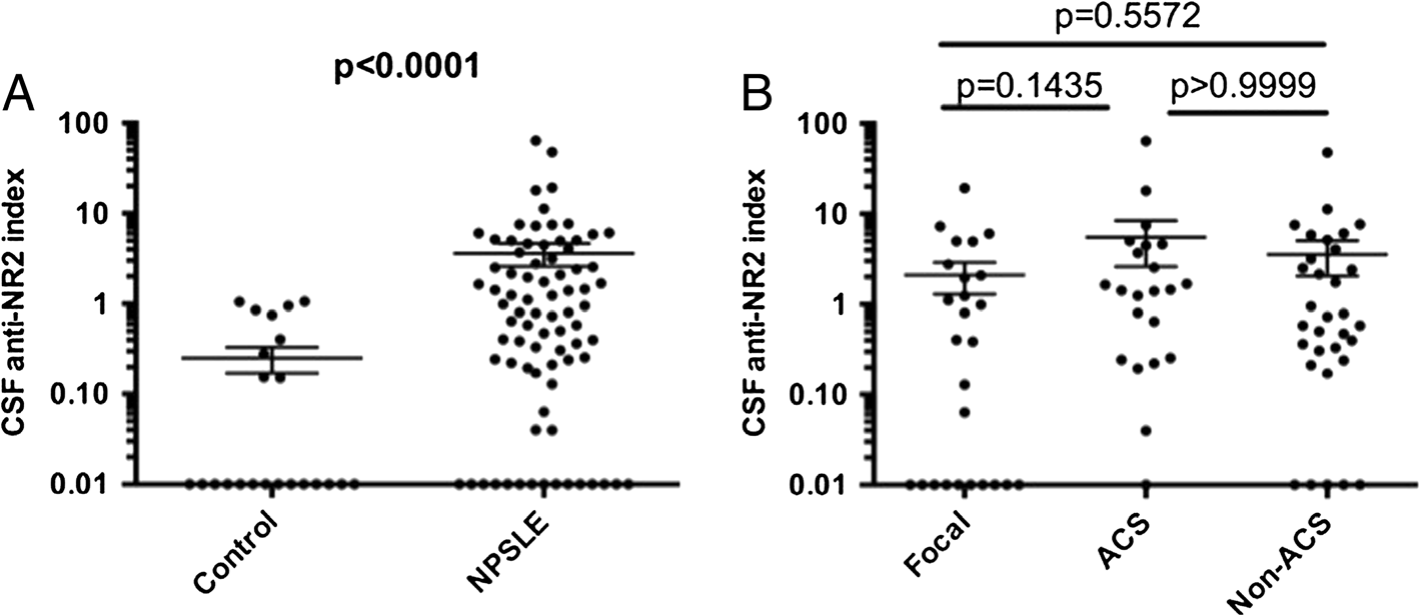
**Intrathecal synthesis of anti-NR2 (CSF anti-NR2 index) in neuropsychiatric systemic lupus erythematosus (NPSLE). (A)** Comparison between NPSLE and non-SLE control, **(B)** comparison among various subtypes of NPSLE. ACS, acute confusional state; Non-ACS, diffuse NPSLE other than ACS, including anxiety disorder, cognitive dysfunction, mood disorder and psychosis; Focal, focal NPSLE. Statistical analysis was performed by the Mann-Whitney *U*-test **(A)** and by the Kruskal-Wallis test with the Dunn multiple comparison test **(B)**.

We next examined intrathecal anti-NR2 synthesis and BBB function in various subsets of NPSLE, including ACS, non-ACS diffuse NPSLE and focal NPSLE, in order to delineate the mechanism of elevation of CSF anti-NR2. As shown in Figure 2, CSF anti-NR2 index, an indicator of intrathecal anti-NR2 production, was significantly elevated in NPSLE compared with the non-SLE control group. However, there were no significant differences in CSF anti-NR2 index among the three groups of NPSLE, including focal NPSLE, ACS and non-ACS diffuse NPSLE. These results indicate that the elevation of intrathecal synthesis of anti-NR2 is enhanced in NPSLE irrespective of the category or the severity.

**Figure 3:**
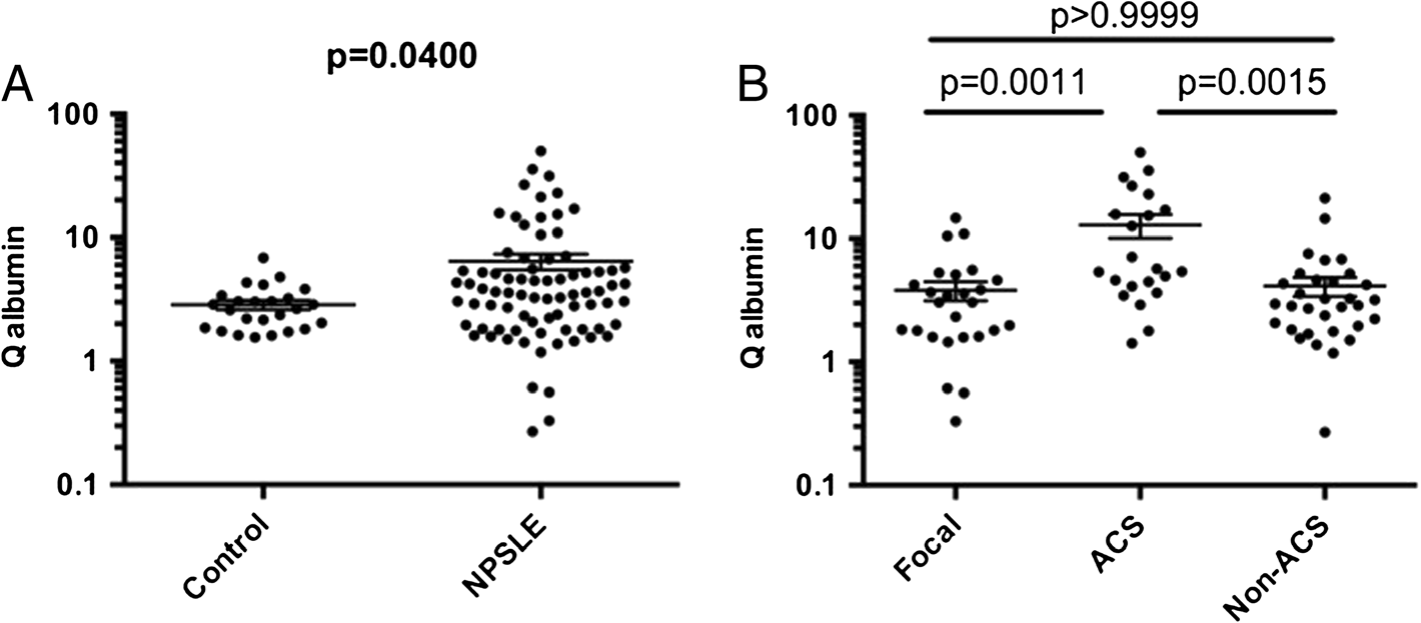
**Blood-brain barrier functions (Q albumin) in neuropsychiatric systemic lupus erythematosus (NPSLE). (A)** Comparison between NPSLE and non-SLE control, **(B)** comparison among various subtypes of NPSLE. ACS, acute confusional state; Non-ACS, diffuse NPSLE other than ACS, including anxiety disorder, cognitive dysfunction, mood disorder and psychosis; Focal, focal NPSLE. Statistical analysis was performed by the Mann-Whitney *U*-test **(A)** and the Kruskal-Wallis test with the Dunn multiple comparison test **(B)**.

**Figure 4:**
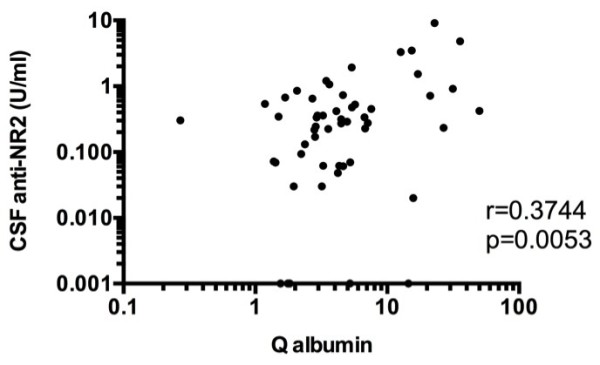
**Correlation between cerebrospinal fluid (CSF) anti-NR2 and Q albumin in patients with diffuse neuropsychiatric systemic lupus erythematosus (NPSLE).** Statistical significance was analyzed by the Spearman rank correlation test.

On the other hand, Q albumin was also significantly elevated in NPSLE compared with in non-SLE control group (Figure 3). More importantly, Q albumin was higher in ACS than in focal NPSLE or in non-ACS diffuse NPSLE. Moreover, there was no significant difference in Q albumin between focal NPSLE and non-ACS diffuse NPSLE. Accordingly, when compared with non-SLE control group, Q albumin was not significantly elevated either in non-ACS diffuse NPSLE or in focal NPSLE. Finally, CSF anti-NR2 was significantly correlated with Q albumin in patients with diffuse NPSLE (ACS and non-ACS) (Figure 4). The results indicate that BBB damages take place in NPSLE. Moreover, the data also support the conclusion that BBB damage plays an important role in the pathogenesis of ACS, the severest form of diffuse NPSLE, allowing the entry of a larger amount of anti-NR2 from the systemic circulation into the brain.

## Discussion

NMDA receptors are a subgroup of the glutamate receptor family, responsible for the majority of excitatory synaptic transmission in the central nervous system [23,24]. DeGiorgio *et al*. showed that injection of anti-NR2 glutamate receptor binding antibodies (purified antibodies from the sera of SLE patients or one CSF sample from an SLE patient with progressive cognitive decline) into the mouse brain resulted in apoptosis of the neuronal cells without signs of inflammation [22]. Of note, Kowal *et al*. have recently demonstrated that mice induced by antigen to express anti-NR2 have no neuronal damage until breakdown of the BBB takes place [25]. Presumably, an intact BBB prevented the transport of anti-NR2 from the systemic circulation into the brain [25]. Previous studies have disclosed that CSF anti-NR2 is elevated in diffuse NPSLE compared with that in focal NPSLE or in non-SLE controls [14]. In the present study, we have demonstrated that CSF anti-NR2 levels were the highest in ACS, the severest form of diffuse NPSLE, among various types of NPSLE. In fact, the effect of anti-NR2 antibodies on neurons has been shown to be dose dependent [26]. Thus, at low concentrations they alter synaptic function, whereas at higher concentrations they can cause neuronal cell death by apoptosis [26]. It is therefore suggested that the presence of higher concentrations of anti-NR2 within the central nervous system might result in greater neuronal damage, leading to the development of ACS.

It should be pointed out that the non-SLE control group is heterogeneous for a relatively small group. However, it is uniform in that they all belong to non-inflammatory diseases in which no immunological abnormalities are involved [20]. Another limitation of our study is that samples were collected for long terms of about 15 years and stored at -30°C. It would have been better to preserve samples at lower temperatures as low as -80°C in order to avoid protein degradation.

Two mechanisms have been implicated for the elevation of CSF IgG. One is transudation through the damaged BBB from the systemic circulation, and the other is intrathecal synthesis [17,19]. Previous studies disclosed that CSF IgG index, an indicator of intrathecal IgG synthesis, is elevated in NPSLE [18,20]. Accordingly, CSF anti-NR2 index was elevated in diffuse NPSLE compared with that in non-SLE control in the present study. However, there were no significant differences in CSF anti-NR2 index between ACS and non-ACS diffuse NPSLE. Therefore, the elevation of CSF anti-NR2 levels in ACS compared with that in non-ACS diffuse NPSLE cannot be accounted for by the increased intrathecal synthesis of anti-NR2. It should be underscored that the elevation of CSF IgG index was not confined to diffuse NPSLE [18,20]. Thus, it is conceivable that CSF IgG index might be elevated in focal NPSLE as well as in ACS and non-ACS NPSLE in our series of patients. Of note, patients with higher CSF IgG index showed CSF oligoclonal IgG bands [18,19]. Again, the presence of CSF oligoclonal IgG bands was not confined to diffuse NPSLE [18]. Taken together, it is strongly suggests that the enhanced intrathecal IgG production might not be specific for diffuse NPSLE, but a may be a common feature in NPSLE.

BBB function has been evaluated by Q albumin, because albumin is produced exclusively in the liver and its presence in CSF is totally dependent on its transudation from the systemic circulation [17,19]. Q albumin values in most patients with NPSLE were found to be below 9.0, the upper normal value of control subjects [18,20]. However, it has not been clarified whether there might be any difference in Q albumin among various types of NPSLE. The results in the present study have disclosed that Q albumin was significantly elevated in NPSLE compared with that in non-SLE controls. More importantly, Q albumin was shown to be apparently higher in ACS than in non-ACS diffuse NPSLE or in focal NPSLE. By contrast, there was no significant difference in Q albumin between non-ACS diffuse NPSLE and focal NPSLE. The data thus indicate that the damage in BBB is a critical factor for the development of ACS. Thus, since there was no significant difference in serum anti-NR2 levels among the 3 groups of NPSLE (data not shown), the damage in BBB is considered to play a critical role in the elevation of CSF anti-NR2 levels in ACS. In fact, CSF anti-NR2 levels were significantly correlated with Q albumin in diffuse NPSLE (ACS and non-ACS) in the present study.

A number of studies have revealed that CSF IL-6 is elevated in patients with NPSLE, and is useful for its diagnosis [5-10]. However, the mechanism of the elevation of CSF IL-6 has not been clarified. Notably, CSF IL-6 was also most markedly elevated in ACS, where CSF anti-NR2 levels were the highest (data not shown). Recent studies have shown that anti-NR2 binds the surface of endothelial cells and enhances their production of proinflammatory cytokines, IL-6 and IL-8, through activation of NFkB [27]. On the other hand, it should be noted that the expression of mRNA for IL-6 was shown to be enhanced in granular neurons in the hippocampus of an SLE patient with diffuse NPSLE [28]. Therefore, it is suggested that interactions of neuronal cells with neuron-reactive autoantibodies, such as anti-NR2, might involve the production of IL-6 by neuronal cells. Further studies are required to confirm this point.

The mechanism of BBB damage in ACS remains to be elucidated. It should be pointed out that anti-ribosomal P enhances the capacity of activated human monocytes to produce IL-6, TNF-α and vascular endothelial growth factor [29,30], which can alter the integrity of the BBB [31-34]. However, neither serum anti-NR2 nor serum anti-ribosomal P was correlated with Q albumin in our series of patients (data not shown). As a variety of autoantibodies are expressed in SLE, it is possible that there might be such autoantibodies other than anti-NR2 or anti-ribosomal P that might cause damage to the BBB. Further studies would be required to identify the whole spectrum of autoantibodies that might cause BBB damage.

## Conclusion

The current studies examined the BBB function and intrathecal synthesis of anti-NR2 in relation to the severity of diffuse NPSLE. CSF anti-NR2 levels and Q albumin were significantly higher in ACS than in non-ACS diffuse NPSLE (anxiety disorder, cognitive dysfunction, mood disorder and psychosis) or in focal NPSLE, whereas there was no significant difference in CSF anti-NR2 index among the three groups. CSF anti-NR2 levels were significantly correlated with Q albumin in diffuse NPSLE. These results support the conclusion that the severity of BBB damages plays a crucial role in the development of ACS, the severest form of diffuse NPSLE, through the accelerated entry of larger amounts of anti-NR2 into the central nervous system.

## Abbreviations

ACR: American College of Rheumatology; ACS: acute confusional state; anti-NR2: antibodies reactive with NMDA receptor NR2 subunit on neuronal cells; BBB: blood-brain barrier; CSF: cerebrospinal fluid; ELISA: enzyme-linked immunosorbent assay; HSA: human serum albumin; IgG: immunoglobulin G; IL: interleukin; NMDA: N-methyl-D-aspartate; NPSLE: neuropsychiatric systemic lupus erythematosus; SLE: systemic lupus erythematosus; TNF: tumor necrosis factor.

## Competing interests

The authors declare that they have no competing interests.

## Authors’ contributions

SH: conception and design, data collection and analysis, manuscript drafting and writing and final approval of the manuscript. YA: data collection and analysis, critical revision and final approval of the manuscript. TYA: data collection and analysis, critical revision and final approval of the manuscript. TYO: data collection and analysis, critical revision and final approval of the manuscript. All authors read and approved the final manuscript.
